# Evaluation of the efficacy and safety of toceranib phosphate in cats with macroscopic mammary adenocarcinoma

**DOI:** 10.1177/1098612X241256473

**Published:** 2024-08-30

**Authors:** Isabel Del Portillo Miguel, Laura Blackwood, Elisa Maiques, Ignacio Pérez Roger, Enric Poch Jiménez, Juan Borrego

**Affiliations:** 1 Hospital for Small Animals, The Royal (Dick) School of Veterinary Studies, The University of Edinburgh, UK; 2 Departamento de Ciencias Biomédicas, Facultad de Ciencias de la Salud, Universidad Cardenal Herrera-CEU, CEU Universities, Alfara del Patriarca, Valencia, Spain; 3 Oncology Service, Hospital Aúna Especialidades Veterinarias, IVC-Evidensia, Paterna, Spain

**Keywords:** Toceranib phosphate, feline cancer, feline mammary tumour, chemotherapy

## Abstract

**Objectives:**

Mammary tumours in cats are biologically aggressive. The standard of care relies upon wide surgical resection. Chemotherapy has been described in the macroscopic disease setting; however, limited efficacy has been shown. The aim of this study was to assess the efficacy of toceranib phosphate in macroscopic feline mammary tumours (FMTs).

**Methods:**

A total of 17 cats with cytologically or histopathologically confirmed mammary adenocarcinoma (gross disease) were prospectively enrolled. Toceranib phosphate was administered at a median dose of 2.77 mg/kg (range 2.3–3.2) PO q48 h. No corticosteroids or non-steroidal anti-inflammatory drugs (NSAIDs) were administered. Toxicity was graded according to Veterinary Cooperative Oncology Group–Common Terminology Criteria for Adverse Events (VCOG-CTCAE) v1.1 criteria. The response was assessed after 1 month, following Response Evaluation Criteria In Solid Tumours (RECIST) criteria.

**Results:**

Toxicity was observed in eight cats, with most instances being grade 1 or 2, which were managed with supportive care. Only one cat experienced grade 3 toxicity (anorexia), which resolved after a dose reduction. Clinical benefit was seen in 12 (64.7%) cats and an objective response was seen in six (35.2%) cats. One cat experienced complete response, five had partial response, six had stable disease and five had progressive disease. One cat showed distant progression (malignant pleural effusion) despite continued partial remission of the primary tumour. The median progression-free survival and median overall survival time were 91 days (range 30–158) and 145 days (range 31–234), respectively.

**Conclusions and relevance:**

Toceranib phosphate showed clinical benefit and a good safety profile in advanced or recurrent FMTs, offering a new alternative in the treatment of this disease; however, further prospective and randomised studies are required to further assess its efficacy. Interestingly, one cat developed distant metastases while the primary tumour showed partial response, suggesting that primary tumour and metastatic disease may not sustain the same sensitivity to toceranib.

## Introduction

More than 80% of feline mammary tumours (FMTs) are malignant,^1^ and tubular, tubulopapillary and solid carcinomas are most common.^2–4^ Malignant FMTs are biologically aggressive, with a high metastatic rate to the regional lymph nodes and lungs. Metastasis is a negative prognostic factor,^5–9^ as is tumour size, disease stage,^10,11^ histological grade^8^ and surgical ‘dose’.^1,7,12^

Surgical removal is the preferred first-line treatment for localised FMTs, either alone or in combination with chemotherapy. Bilateral mastectomy significantly increases both median progression-free survival (PFS) and overall survival time (OST) when compared with unilateral mastectomy, with survival extending to 917 days compared with 566 days.^7,12^ Chemotherapy (doxorubicin alone or combined with cyclophosphamide) has been used in macroscopic disease,^13–16^ with objective responses in 50% of inoperable or stage IV tumours (those with distant metastases).^13^,^14^,^17^ Postoperative adjunctive chemotherapy has also been investigated and is often recommended by oncologists,^15^ despite the lack of clear survival benefit.^10^,^16^,^18^–^20^

Toceranib phosphate (Palladia; Pfizer) is a multitarget tyrosine kinase inhibitor (TKI), similar to sunitinib, with activity against KIT receptor, vascular endothelial growth factor receptor (VEGFR), platelet-derived growth factor receptors (PDGFR) and others. Toceranib can also decrease regulatory T cells.^21^ These tyrosine kinases are expressed in mast cell and other tumours in dogs and also in cats.^22–26^ In cats, a clinical response has been demonstrated in squamous cell carcinomas^27,28^ and mast cell tumours.^29,30^ Clinical benefit has also been reported in carcinomas in diverse locations, including vertebral osteosarcoma, chemodectoma and cutaneous melanoma, when treated with toceranib, either as monotherapy or in conjunction with surgery,^31–36^ but not in injection site sarcomas.^37^ Some case series include FMTs, but case numbers were low and specific FMT efficacy was not determined.^30,31^ A recent study showed no difference in survival between patients with stage IV disease (those with measurable metastases) receiving traditional chemotherapy, metronomic chemotherapy and toceranib.^38^

Toceranib is well tolerated in cats.^39^ Reported side effects are mainly gastrointestinal, most often Veterinary Cooperative Oncology Group (VCOG) grades 1 and 2 (anorexia, vomiting and diarrhoea), similar to those described in dogs.^27,28,30,31,37,39^ Severe gastrointestinal side effects were uncommon and managed by temporarily discontinuing the drug or reducing the dose or frequency of administration. There may be a higher incidence of hepatotoxicity in cats compared with dogs.^30,39^

The primary aim of this prospective study was to evaluate the effectiveness of oral toceranib in treating macroscopic FMTs. The secondary goal was to examine the safety of toceranib in this specific population.

## Materials and methods

Cats presenting to the referral hospital Instituto Veterinario de Oncología Comparada (IVOC, Valencia, Spain) with a measurable, primary mammary adenocarcinoma confirmed histologically or cytologically, with or without metastasis, between December 2012 and 2015 were enrolled. Primary tumours were categorised as de novo (newly detected) or recurrent, and patients with metastatic disease were included. Cats were either not considered surgical candidates or the client declined surgery. Client consent was required for enrolment. This study did not require institutional ethical approval, as established internationally recognised ‘best practice’ was followed, and clinical decision-making and patient management were not altered by recruitment.

Histopathological classification (conducted in a single laboratory by non-board-certified pathologists) followed the Elston and Ellis grading system.^40,41^ Cases diagnosed using cytology were classified as carcinoma if at least 30% of the cells showed more than three criteria of malignancy.^42–44^ Samples from lymph nodes were classified as metastatic if one or more clusters of epithelial cells with criteria of malignancy were detected.

The initial assessment of the patients (within 7 days before the start of therapy) included the following: physical examination; measurement of the maximum diameter of the primary lesion; full abdominal ultrasound; three-view thoracic radiography or thoracic CT; complete blood count (CBC); biochemistry (minimum alanine aminotransferase [ALT], alkaline phosphatase [ALP], creatinine, urea, glucose and total solids [TS]); and urine analysis (pH, density, glucose, ketone bodies, nitrites, leukocytes, bilirubin, protein). The urine protein:creatinine (UPC) ratio was measured if proteinuria was detected. Fine needle aspirates (FNAs) were taken from abnormal lymph nodes or suspected metastatic lesions detected on physical examination or imaging techniques. Cats were excluded for the following reasons: if they had received chemotherapy, corticosteroids or non-steroidal anti-inflammatory drugs (NSAIDs) in the 2 weeks before enrolment; had haematological or biochemical abnormalities likely to impact survival or preclude administration of toceranib; or were systemically unwell or had non-mammary tumour related estimated life expectancy >2 months. Cats receiving other chemotherapy treatment or radiation therapy during toceranib treatment were excluded. However, supportive treatments (including antibiotics, anti-inflammatories and corticosteroids) were permitted as needed.

Toceranib phosphate was prescribed at a starting dose between 2.3 and 3.2 mg/kg q48h, at the discretion of the prescribing clinician, and compounding was allowed.

Information collected included signalment, weight, reproductive status (including spayed date), previous hormonal treatments, location and size of the tumour (maximum diameter, cm), previous tumour treatments, and histopathology and cytology reports. Best response was assessed as per modified Response Evaluation Criteria In Solid Tumours (RECIST)^45^ (Table 1), and toceranib-related toxicity was assessed according to VCOG–Common Terminology Criteria for Adverse Events (VCOG-CTCAE) criteria^46^ (Table 2). When cats presented with two tumours, the one with the longest diameter was considered for the RECIST response.^45^ Dose reductions or delays, rescue treatment, dates of progression and death, and cause of death were also collected.

**Table 1:** Modified Response Evaluation Criteria In Solid Tumours (RECIST) criteria as applied to 17 cats with macroscopic mammary tumours^45^

CR	Disappearance of all target lesions, or normalisation of lymph nodes
PR	At least 30% reduction in the longest diameter of target lesion
SD	Less than 30% reduction (or 20% increase) in the longest diameter of target lesion
PD	Either the appearance of one or more new lesions or at least a 20% increase in the diameter of target lesion

CR = complete response; PD = progressive disease; PR = partial response; SD = stable disease

**Table 2:** Veterinary Cooperative Oncology Group–Common Terminology Criteria for Adverse Events for the most common in the study^46^

Adverse event	Grade 1	Grade 2	Grade 3	Grade 4	Grade 5
Neutropenia (/μl)	1500 to <LLN	<1000–1499	500–999	<500	Death
ALT	>1.5 times upper limit	>1.5–4.0 times upper limit	>4.0–10 times upper limit	>10 times upper limit	Death
Vomiting	Lasting ⩽24 h that resolves with or without the use of medication and/or parenteral fluids	Lasting >24 h	Requires hospitalisation, interfering with activities of daily living (eating, drinking, sleeping, defecating and urinating)	Life-threatening (eg, haemodynamic collapse)	Death
Anorexia	Lasting <48 h	Lasting 2–3 days	Duration of 3–5 days; associated with significant weight loss (⩾10%) or malnutrition; IV fluids, tube feeding or force feeding indicated	Duration of >5 days; life-threatening consequences; total parenteral nutrition indicated	Death

ALT = alanine aminotransferase; IV = intravenous; LLN = lower limit of normal

Cats were reassessed (physical examination and CBC) 2 weeks after starting toceranib. The response was assessed after 1 month, repeating physical examination, measurement of the mass and imaging tests as performed on the initial assessment. At this visit, CBC, biochemistry and urine analysis were repeated to assess for toxicity. Cats that showed progressive disease (PD) were offered rescue treatments (alternative medical therapies, at the discretion of the clinician), while cats that responded or had stable disease (SD) continued on toceranib with a monthly assessment, including physical examination, CBC, biochemistry and urine analysis. Imaging tests were repeated every 3 months. Clinical benefit was complete response (CR), partial response (PR) or SD for at least 2 months.

Dose-limiting toxicities (DLTs) were those graded as 3 or 4, which were refractory to supportive treatment (maropitant or mirtazapine for gastrointestinal toxicity) or lasted >7 days. If cats experienced DLTs, toceranib treatment was temporarily suspended (usually for 5–7 days) and/or the dose (minimum 2.3 mg/kg) and/or the frequency of administration were reduced, at the clinician’s discretion.

Statistical analysis was performed using commercially available software (GraphPad Prism version 7.0; GraphPad Software).

The Kaplan–Meier product-limit method was used to determine median PFS (MPFS) and median OST (MOST). PFS was defined as the time from inclusion to the study (starting toceranib) to disease progression, while OST was defined as the time from inclusion in the study to death.

## Results

A total of 23 cats were presented with macroscopic primary mammary adenocarcinoma (confirmed histologically or cytologically). Two were excluded: one due to stage IV chronic kidney disease (CKD); the other due to concurrent chemotherapy. Four eligible cats were not enrolled as their owners declined treatment. In total, 17 cats were included. A total of 16 owners declined surgery due to either financial constraints or previous negative experiences that made them unwilling to consider surgical procedures or anaesthesia. One cat was not considered a surgical candidate due to metastases to the suprasternal lymph node. Population characteristics and response data are summarised in Table 3.

**Table 3:** Clinical characteristics and response of 17 cats with macroscopic mammary carcinoma treated with toceranib

Case	Age (years)	Breed	Previous surgery	Presentation	Primary mass size (cm)	Histopathological grade	Ulceration	Metastasis at presentation	Previous chemotherapy	Dose toceranib (mg/kg)	Response (of primary lesion)
1	8	DSH	No	De novo	2.3	II	No	No	No	2.8	SD
2	12	Persian	No	De novo	3.7	II	Yes	No	Docetaxel	2.3	PR
3	11	DSH	Yes	Recurrence	4	N/A	No	No	No	3.0	PD
4	10	DSH	Yes*	De novo	1.8	III	No	No	No	2.6^†^	SD
5	16	DSH	Yes	Recurrence	5.1	N/A	Yes	No	Doxorubicin	2.6^†^	PR
6	9	Siamese	Yes	Recurrence	3.2	N/A	Yes	No	No	3.2	PD
7	10	DSH	No	De novo	4.1	II	No	No	Docetaxel	2.3	SD
8	12	DSH	Yes*	De novo	3.3	III	No	No	No	2.7	SD
9	8	DSH	Yes	Recurrence	2.1	III	Yes	No	No	3.1	PR
10	14	DSH	No	De novo	2.6	III	No	No	No	2.8	PD
11	8	Siamese	Yes*	De novo	1.5	N/A	No	No	No	2.6	CR
12	14	DSH	No	De novo	1.7	III (post morten)	No	No	No	2.5	SD
13	8	DSH	No	De novo	2.1	III	No	Axillary and sternal LN	No	3.0	PR
14	12	DSH	Yes*	De novo	2.8	II	No	No	No	2.9^†^	SD
15	11	Siamese	Yes*	De novo	4.3	N/A	No	Axillary LN	Doxorubicin	3.1	PR
16	9	DSH	No	De novo	2.7	III	No	Inguinal LN	No	2.4	PD
17	10	DSH	Yes*	De novo	2.9	II	No	No	no	2.8	PD

a *Previous surgery for a different mammary tumour

† Cats for which toceranib phosphate was compounded

CR = complete response; DSH = domestic shorthair; LN = lymph node; N/A = not available; PD = progressive disease; PR = partial response; SD = stable disease

The median age of the cats was 10.6 years (range 8–16 years). All cats were female with 12 intact and five spayed cats (four had been spayed after 2 years of age; in the fifth cat, the date was unknown). In total, 13 cats were domestic shorthairs, three were Siamese and one was Persian. The median body weight was 3.8 kg (range 2.1–6.2). No cat had received hormonal treatments, including exogenous oestrogen, progesterone or combined treatments.

Four cats had recurrent primary tumours after surgery and six cats had de novo (newly detected) tumours after previous surgery for an FMT in another site (6/13, 46%). Four of the cats with de novo tumours had contralateral tumours after unilateral chain mastectomy, and two in the same chain after local mastectomies. The remaining sevn cats did not have a history of previous mammary tumours. Two cats presented with two tumours. In nine cats, the tumour was in the caudal mammary glands; in six cats, the tumour was in the cranial mammary glands; and in two cats, there were lesions in both. The median maximum tumour diameter was 2.95 cm (range 1.5–5.1). Four cats presented with ulcerated tumours, three of which were recurrent. Two of these cats were receiving cefovecin (Convenia; Zoetis), while the other two were treated with topical cleaning/disinfection.

A definitive diagnosis was achieved by punch biopsy in nine cats and wedge biopsy in two. There were no post-biopsy complications. Six cats were diagnosed with grade III adenocarcinoma, while five cats had grade II adenocarcinoma. Four cats had tubulopapillary adenocarcinomas and four had solid adenocarcinomas; in the remaining case, no subtype was assigned. In one case initially diagnosed by cytology, a grade III adenocarcinoma was confirmed at necropsy. Three cats presented with lymph node metastases, affecting the axillary and sternal nodes in one case, the axillary in the second and the inguinal in the third. No cats had pulmonary metastases at presentation: one cat had a thoracic CT that showed enlargement of the axillary lymph nodes. An abdominal ultrasound revealed no intra-abdominal metastasis in any of the cats.

CBC was performed in all 17 cats. Of them, four (23.5%) had mild anaemia (packed cell volume 25–35%, reference interval [RI] 30.3–52.3). White cell counts were in the range of 4.5–22.3 ×10^3^/μl (RI 5.1–17.2) with four cats having leukocytosis. One cat had a slight elevation of ALT (88 UI/l, RI 12–30), another had elevated urea (35 mg/dl, RI 5.7–12.9) and two cats had stage 2 CKD (International Renal Interest Society classification).

Four cats had previously received chemotherapy and received toceranib after disease progression: two cats had disease progression after treatment with doxorubicin (1 mg/kg IV every 3 weeks); and two cats had progression after docetaxel (2.25 mg/kg IV every 3 weeks). In all four cases, PD was recorded before the third planned chemotherapy treatment. The median administered dose of toceranib was 2.77 mg/kg PO q48h (range 2.3–3.2). In three cats, toceranib was compounded. No cats received corticosteroids or NSAIDs during toceranib treatment. The overall response rate was 35.2% (6/17), with a clinical benefit in 64.7% (12/17) of cases. One cat experienced CR, five PR), six SD for 2 months or more and five experienced PD. Three cats that experienced PR also experienced improvement in ulceration of primary masses. In one case (cat 15), the response was assessed by CT 30 days after starting toceranib, achieving PR for the primary tumour. Moreover, the assessment of the metastatic axillary lymph node was consistent with SD.

Eight cats (47%) had side effects, with only one cat showing DLTs (Table 3). Three cats had grade 1 neutropenia and two had grade 2 liver enzyme elevations during the first 3 months on toceranib. Both cases with liver toxicity were managed by decreasing the frequency of toceranib administration to twice per week. One cat experienced grade 2 vomiting, and two experienced grade 1 and grade 3 anorexia, respectively. These cats were given supportive care with a course of maropitant and mirtazapine, and clinical signs resolved.

**Figure 1:**
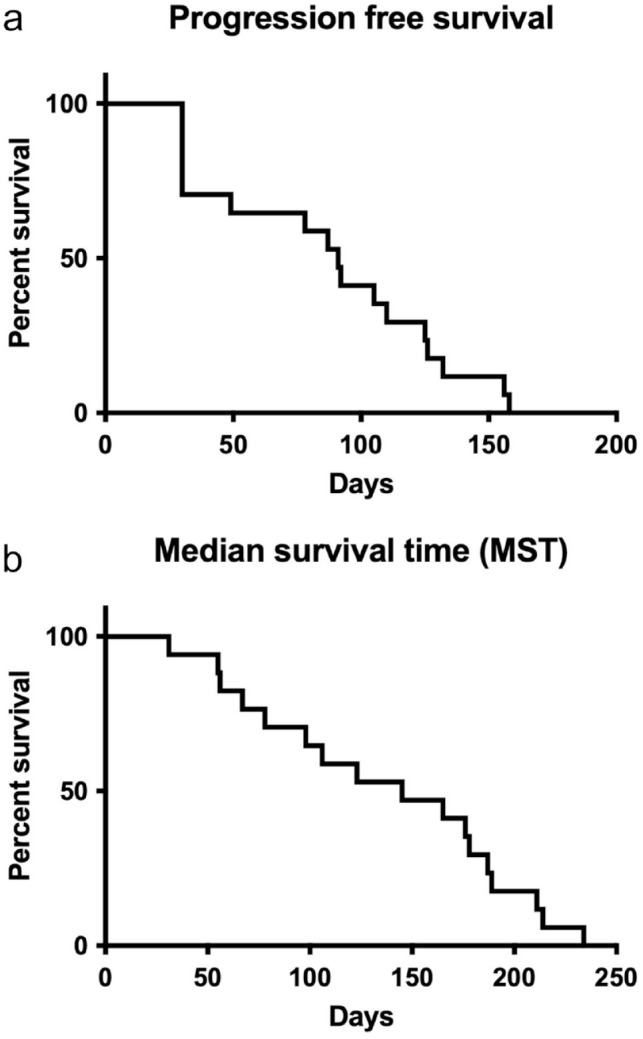
Kaplan–Meier survival analysis for median (a) PFS and (b) OST for 17 cats with mammary adenocarcinoma treated with toceranib phosphate. The median PFS was 91 days and the median OST was 147 days. OST = overall survival time; PFS = progression-free survival

The MPFS and MOST were 91 (range 30–158) and 145 days (range 31–234), respectively. PFS and OST for the cats with metastases at presentation were 125, 128 and 30 days and 234, 189 and 78 days, respectively. The Kaplan–Meier curves are shown in Figure 1.

One cat developed acute respiratory distress secondary to pleural effusion, detected on day 43, despite maintaining PR in the primary tumour. The cytology of the pleural effusion was consistent with epithelial malignancy. Concurrent pulmonary metastases were not detected on radiographs, but the presence of the fluid limited interpretation. No pulmonary metastases had been detected at the initial staging.

Rescue treatments were offered to all cats when PD occurred. Four cats received docetaxel (2.25 mg/kg IV every 3 weeks) and one cat received doxorubicin (1 mg/kg IV every 3 weeks). One of the cats that received docetaxel had a PR, two cats achieved SD and one had PD. The cat treated with doxorubicin received a single dose without response.

All cats were included in the survival analysis: none were alive at 1 year and all died due to FMT progression. In seven cases, the cause of death was pulmonary metastases, and in two further cases malignant pleural effusion (cytologically confirmed). In five cases, the progression of local primary disease occurred with uncontrollable local inflammation, infection or bleeding, triggering euthanasia. Three cats were euthanased due to decreased quality of life (lethargy and anorexia). Overall, 14 cats were euthanased and three died naturally after being diagnosed with pulmonary metastasis.

## Discussion

To the authors’ knowledge, this is the first prospective study assessing the response and toxicity profile of toceranib in macroscopic FMTs. Data on TKIs in FMTs are limited, but expression of VEGF and VEGFR has been reported,^47,48^ supporting the use of toceranib. Toceranib has also been used in other non-mast cell tumours of various types, predominantly carcinomas.^27,28,30–33^

The median age of the cats in this study was 10.6 years, similar to previous cohorts.^10,16,38^ Most cats were intact or spayed when adults, again as described previously.^10,16^ It is known that early neutering reduces the risk of developing mammary tumours.^49^ No cats in this study received hormonal treatments such as exogenous progestins or oestrogen–progestin combinations, a practice that has largely ceased due to the relationship between the administration of these drugs and mammary cancer.^50,51^

Overall, cats included in the study had advanced disease, with 4/17 (23.5%) cats presenting with local recurrences after previous surgeries and 3/17 (17%) cats having metastatic disease. Moreover, 4/17 (23.5%) cats had received chemotherapy previously. The advanced stage and previous treatments may have had a negative impact on the response to toceranib.

Of the 13 cats with de novo tumours, six (46%) had previously had mammary tumours in other mammary glands; this finding supports the recommendation for bilateral mastectomy in FMT, and may also reflect a reluctance to recommend this surgery in primary care practice. These de novo tumours were either truly independent tumours or metastases from previous tumours. The lymphatic drainage system of the mammary glands in cats is different from dogs, with connections between all the glands.^52^ Because of this, resection of at least one mammary chain (and ideally both) is recommended,^1^ and bilateral chain mastectomy prolongs the disease-free interval.^11^

True recurrence was also a common finding, supporting the recommendations of wide surgical margins (including at least 2 cm in the periphery and a deep fascial plane).^1,15^

The distribution of tumours in this study was similar between the caudal and cranial mammary glands, and only two patients had multiple tumours at diagnosis. However, a higher incidence of mammary tumours arising from the caudal glands^1^ and a high frequency of multiple tumours at initial diagnosis has been reported previously.^15,53^

The size of the mammary tumours is a well-established prognostic factor, and the median size in our study was 2.95 cm, which is higher than in other studies,^10,11,16^ in keeping with a population with advanced disease. Seven cats (41%) had a tumour exceeding 3 cm, a size clearly associated with a poor prognosis.^1,54,55^ Ulceration has also been associated with poorer prognosis and four (23%) cats in the study showed ulceration, similar to the existing literature.^55^

Similar to human breast cancer, where cytology is a reliable method to achieve a definitive diagnosis (77–99%), FNAs are reliable in cats, with good concordance between cytology and histopathology.^56^ For this reason, cytology was accepted as a diagnostic method and published cytological criteria were applied.^56^

Most tumours were histologically high-grade, as expected based on the literature.^42,57^ None were diagnosed as inflammatory mammary carcinoma.

In three (17%) cats, metastatic lymph nodes were also diagnosed based on cytology at the time of diagnosis. Cats with metastatic lymph nodes have shorter free survival time,^7–9,58^ and approximately 25% of the cats with mammary tumours have lymph node metastasis,^1^ affecting mainly the axillary and inguinal lymph nodes. Sternal lymph nodes may also be affected in up to 30% of cats.^1^ In this study, lymph nodes were only aspirated if they were enlarged on physical examination or abnormal on imaging; therefore, the rate of metastasis may be underestimated. In addition, in up to 22% of metastatic carcinoma lymph nodes, cytology may not detect cancer cells that are obvious in histopathological studies.^59^

Of the cats, 35.2% achieved an objective response, while 64.7% experienced clinical benefit, supporting the hypothesis that toceranib has a positive effect in the treatment of FMTs. Case numbers were too low to allow for meaningful statistical analysis, but there was no apparent correlation between tumour size, stage or grade with likelihood of response. The individual responses observed in this study may reflect the antiangiogenic or antitumour properties of the drug, or a combination of both effects.^60^

These response rates are comparable with those described for traditional chemotherapy (in a gross disease setting), where reasonable objective response rates PR and CR of 45–64% have been reported in some studies.^13,14,17^ However, others report lower response rates or only SD, with clinical benefit in 26–59.9% of cases.^33,61^ Patient numbers are low in all studies, but overall the biological efficacy appears broadly similar to that of toceranib in advanced macroscopic canine mammary tumours.^62^ In addition, a recent study^38^ of 10 cats with macroscopic mammary tumours treated with toceranib also suggested toceranib is a reasonable treatment option, though specific response was not evaluated. This response in the macroscopic setting suggests that toceranib might be effective as adjuvant therapy in microscopic disease; however, further prospective and randomised studies are required, as metastatic disease may not exhibit the same sensitivity as the primary tumour.

Sunitinib, a drug almost identical in its structure to toceranib, is used as monotherapy in ‘triple negative’ human breast cancer and has shown response rates in the range of 3–55%. However, first-line therapy does not improve outcomes compared with traditional chemotherapy.^63,64^ In addition, the response to sunitinib is worse in patients who have previously received traditional chemotherapy rescue treatments.^63^ In this study, four cats had previously received traditional chemotherapy, which may have altered the response to toceranib.

Three cats achieved a PR with the improvement of ulceration, likely to be of significant clinical benefit, and a similar response has been described in humans with sunitinib.^64^

One cat (with PR of an ulcerated tumour) developed a malignant effusion before progression of the primary lesion. This development of metastatic disease alongside continued response of the primary tumour has been described in several human tumours.^65,66^ Differences in response in primary and metastatic tumours may reflect a possible non-uniform distribution of TKIs in different organs, or different expression of proteins between primary tumours and metastases as described in human breast cancer.^67^

In human medicine, when some antiangiogenic treatments, particularly sunitinib, are given after surgical removal of the primary tumour, progression of metastasis can accelerate.^68^ This may reflect the selection and overgrowth of subpopulations of cancer cells resistant to the treatment simply by activating pathways of proangiogenic factors.^69^ This may explain the differences in the response of primary and metastatic lesions in the current study.

Toceranib was well tolerated, with only one cat showing DLT (anorexia). Of the cats, 47% experienced mild adverse events; these were mainly haematological and gastrointestinal events, managed by decreasing the administration to twice per week. Toxicities were similar to those in previous publications;^27,28,30,31,38,39^ however, azotaemia was not reported in this study. As in other studies, the cats that experienced an increase in ALT did not show any clinical signs of hepatopathy.^30^ Although routine monitoring of the blood pressure and UPC ratio was not performed, none of the cats showed proteinuria on urine analysis.

The MPFS of 91 days is very similar to that reported in a study assessing the response to docetaxel in macroscopic FMT (84 days).^61^ A study assessing different medical options, including toceranib, metronomic chemotherapy and traditional chemotherapy, reported a MPFS of only 50 days;^38^ however, the MPFS was not calculated specifically for the group treated with toceranib.^38^ Further, the MOST was only 63 days for the FMT group treated with toceranib, perhaps due to the patients all having stage IV disease. This is shorter than both the MPFS and MOST (145 days) in the current study.

The present study has some limitations. These include the small sample size, inconsistencies in patient enrolment criteria (with the inclusion of both treatment-naive and previously treated cats), and the variation in diagnostic and imaging techniques and for evaluating treatment response. In addition, the use of compounded toceranib represents a potential limitation, given the absence of studies on its efficacy and noted variability in the potency of compounded chemotherapy drugs,^70–72^ which could affect the drug’s effectiveness. Future prospective studies could address these issues through a larger sample size, standardised enrolment and staging criteria, and monitoring and follow-up processes.

## Conclusions

Toceranib is a reasonable therapeutic option in cats with advanced or recurrent mammary tumours, showing clinical benefit and a good toxicity profile. Further prospective and randomised studies are required to further assess its efficacy in this setting. Moreover, this cohort includes the first report of a cat showing a different response to toceranib in the primary tumour and metastatic disease suggesting that primary tumours and metastatic disease may not show the same sensitivity to toceranib.
